# Transformation of macrophages into myofibroblasts in fibrosis-related diseases: emerging biological concepts and potential mechanism

**DOI:** 10.3389/fimmu.2024.1474688

**Published:** 2024-09-25

**Authors:** Xiujun Li, Yuyan Liu, Yongjun Tang, Zhaoyi Xia

**Affiliations:** ^1^ Health Science Center, Chifeng University, Chifeng, China; ^2^ Rehabilitation Medicine College, Shandong Second Medical University, Jinan, China; ^3^ Department of Emergency, Affiliated Hospital of Chifeng University, Chifeng, China; ^4^ Department of Library, Children’s Hospital Affiliated to Shandong University, Jinan, China; ^5^ Department of Library, Jinan Children’s Hospital, Jinan, China

**Keywords:** macrophages, myofibroblasts, macrophage-to-myofibroblast transformation (MMT), TGF-β signaling pathway, fibrosis

## Abstract

Macrophage-myofibroblast transformation (MMT) transforms macrophages into myofibroblasts in a specific inflammation or injury microenvironment. MMT is an essential biological process in fibrosis-related diseases involving the lung, heart, kidney, liver, skeletal muscle, and other organs and tissues. This process consists of interacting with various cells and molecules and activating different signal transduction pathways. This review deeply discussed the molecular mechanism of MMT, clarified crucial signal pathways, multiple cytokines, and growth factors, and formed a complex regulatory network. Significantly, the critical role of transforming growth factor-β (TGF-β) and its downstream signaling pathways in this process were clarified. Furthermore, we discussed the significance of MMT in physiological and pathological conditions, such as pulmonary fibrosis and cardiac fibrosis. This review provides a new perspective for understanding the interaction between macrophages and myofibroblasts and new strategies and targets for the prevention and treatment of MMT in fibrotic diseases.

## 1 Introduction

**Figure 1:**
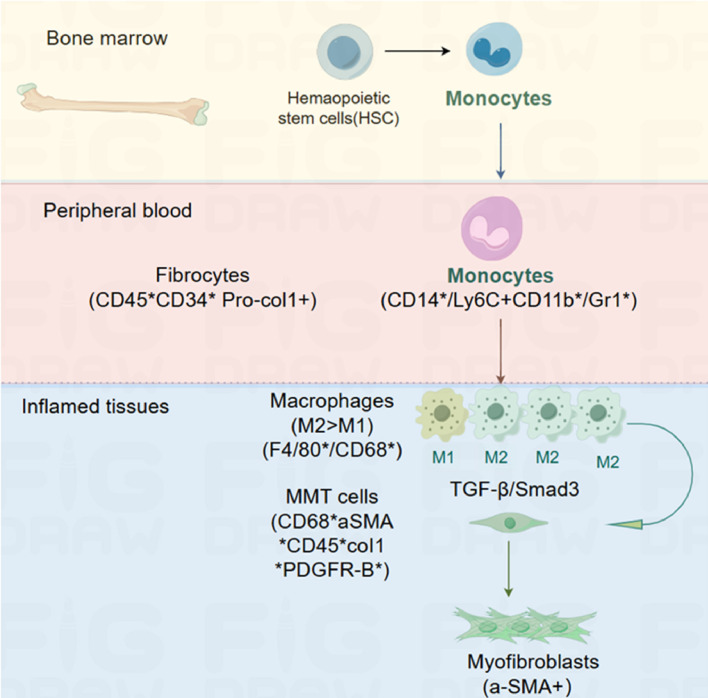
MMT in tissue fibrosis.

Macrophage-myofibroblast transformation (MMT) describes how macrophages from circulating monocytes originating in the bone marrow transform into myofibroblasts and contribute to fibrosis (1, 2). The term was coined by Nikolic-Paterson et al. In 2014 (3). MMT is a newly discovered mechanism that occurs in damaged tissues undergoing fibrosis; the study of MMT relies on the detection of intermediate cells that co-express macrophage markers, such as CD68, and myofibroblast markers, such as α-smooth muscle actin (SMA) (4, 5). Hematopoietic stem cells (HSC) can differentiate into monocytes in the bone marrow. Blood monocytes entering the injured tissue can differentiate into an M2 pro-fibrotic phenotype, either directly or via an M1 pro-inflammatory phenotype. TGF-β/Smad3 signaling drives macrophage transition into collagen-producing α-SMA myofibroblasts via MMT (6) (**Figure 1**).

MMT is considered one of the essential mechanisms for the origin of myofibroblasts in solid organs (7–11). Experimental models of fibrosis, including lung fibrosis, renal fibrosis following transplantation or ureteric obstruction, and post-myocardial infarction fibrosis, have demonstrated MMT as an additional source of myofibroblasts (2, 3, 6, 12, 13). Wang et al. (1) also observed the occurrence of MMT, which contributes to interstitial fibrosis in case of human chronic active renal allograft injury. This was identified through the co-expression of macrophage markers (CD68 or F4/80) and myofibroblast markers (α-SMA). Similarly, Little et al. (14) demonstrated the presence of MMT in the subretinal fibrotic lesions, which ultimately led to subretinal fibrosis. Increasing evidence supports the role of macrophages in promoting fibrosis through their transformation into myofibroblasts, a process known as the MMT (15). Several signaling pathways, including TGF-β1/Smad, Notch, and Wnt signaling pathways, including are involved in MMT (3). It is worth noting that several studies have specifically highlighted the promotion of MMT by the TGF-β1/Smad2/β-catenin signaling pathway (3, 16–19).

This review provides an update on current advancements in MMT and summarizes recent evidence and mechanisms of MMT in fibrosis. Furthermore, we discussed the significance of MMT in physiological and pathological conditions. Under physiological conditions, MMT may participate in tissue repair and wound healing, which helps restore the structure and function of tissues. Under pathological conditions, excessive transformation may lead to the occurrence and development of fibrotic diseases, such as pulmonary fibrosis (PF) and cardiac fibrosis. Understanding this phenomenon and its underlying signal pathway would be beneficial in finding therapeutic targets for fibrosis disease.

## 2 Overview of macrophage

Macrophages were first described by Elie Metchnikoff in 1893 when he observed phagocytes attacking and engulfing microbes in starfish challenged by a rose thorn (20). Another significant milestone came in 1924 when Aschoff defined macrophages as a part of the reticulo-endothelial system (21). However, in 1968, Van Furth et al. (22) proposed the mononuclear phagocyte system, challenging the previous definition. According to this system, all macrophages were believed to originate from the terminal differentiation of circulating monocytes. This theory was further supported by other researchers around the world at that time (23–25). However, more recent studies have identified a dual origin of tissue macrophages. It has been found that macrophages can differentiate from circulating monocytes derived from bone marrow stem cells, as well as primitive macrophages derived from the embryonic yolk sac and fetal liver (26, 27). The mononuclear phagocyte system consists of three parts, including monocytes, macrophages, and dendritic cells, with macrophages playing a crucial role within this system (28).

Macrophages are strategically located throughout body tissues, ingesting and processing foreign bodies, dead cells, and debris while recruiting additional macrophages in response to inflammatory signals. These cells are highly heterogeneous cells and have the ability to rapidly change their function in response to local microenvironment signals (29). Macrophages are categorized into subsets based on their anatomical location and functional phenotype (30). Some examples of specialized tissue-resident macrophages include osteoclasts (bone), alveolar macrophages (lung), histiocytes (interstitial connective tissue), and Kupffer cells (liver). It is important to note that there is considerable overlap in the expression of surface markers between different subsets of macrophages (31).

Rather than being discrete and stable subsets, macrophages represent a spectrum of activated phenotypes (32). Classically activated macrophages, also known as M1 macrophages, are involved in host defense against various bacteria, protozoa, and viruses, and they also play a role in anti-tumor immunity. On the other hand, alternatively activated macrophages, or M2 macrophages, possess anti-inflammatory properties and contribute to wound healing. There are also “regulatory” macrophages that can secrete high levels of interleukin-10 (IL-10) upon binding to Fc receptors gamma (33, 34). Macrophages found in the lung (both interstitial and alveoli), peritoneum, liver (Kupffer cells), and brain (microglia) are generally considered to be distinct lineage of macrophages with unique functions (35, 36).

### 2.1 The classification and phenotype of macrophages

Monocytes are regarded as precursor cells of the mononuclear phagocytic system, with macrophages being one of the key members of this cellular system. Within the macrophage population, there exist various subpopulations of macrophages, each with its characteristics and functions.

#### 2.1.1 Classification of organizational sources

The specialization of macrophages in particular microenvironments explains their heterogeneity. Macrophages take different names according to their tissue location, such as osteoclasts (bone), alveolar macrophages (lung), microglial cells (central nervous system), histiocytes (connective tissue), Kupffer cells (liver), and LC (skin). These populations have such highly different transcriptional profiles that they could be considered as many different and unique classes of macrophages (37).

#### 2.1.2 General functional classification

Macrophages can be defined and classified based on their functions, such as phagocytosis and immunity, as well as specific markers like F4/80 and CD68 (38). This classification divides them into:

##### 2.1.2.1 Classically activated macrophages

Classically activated macrophages, or M1 macrophages, are induced *in vitro* by interferon (IFN)-γ and lipopolysaccharide (LPS). They drive a pro-inflammatory response and aid in the elimination of infection. Mainly through the secretion of pro-inflammatory cytokines (such as IL-1, IL-6, TNF-α, etc.) and chemokines, they promote the occurrence and development of inflammatory reactions. They can devour and eliminate foreign pathogens, activate the immune response of T cells, and regulate and promote the Th1 immune response.

##### 2.1.2.2 Selectively activated macrophages

Selectively activated macrophages, known as M2 macrophages, play a role in controlling the immune response and tissue remodeling (39). M2 macrophages encompass a variety of phenotypes that further subdivided into M2a (exposure to IL-4 or IL-13), M2b (induced by immune complexes in combination with IL-1β or LPS), M2c cells (after exposure to IL-10, TGF-β or glucocorticoids) and M2d cells (IL-6, angiogenic adenosineA2A) (40, 41). M2 macrophages inhibit inflammatory reactions and promote tissue repair and wound healing mainly by secreting anti-inflammatory cytokines (such as IL-10) and growth factors (such as vascular endothelial growth factor (VEGF) and TGF-β). They also regulate the Th2 immune response, which is beneficial for disease recovery in the late stage of inflammation.

Stimulated by GM-CSF, IFN-γ, and LPS, M0 macrophages polarize into M1 macrophages. Alternatively, M-CSF, IL-4, IL-13, and immune complexes (IC) stimulation cause the polarization of M0 macrophages to M2 macrophages. Various cytokines further induce M2 macrophages to differentiate into M2a, M2b, M2c, and M2d phenotypes. M1 macrophages are usually associated with inflammation and represent a prototypic subset of pro-inflammatory macrophages (39). In contrast, M2 macrophages are polarized by Th2 cytokines IL-4 and IL-13, among other factors. They are characterized by high levels of anti-inflammatory cytokines and pro-fibrotic factors (39, 42), contributing to matrix deposition and tissue remodeling (43). M2 macrophages are the primary source of TGF-β1, which is widely recognized as a critical cytokine associated with fibrosis (39, 44, 45). M2 macrophages have been found to affect pathological fibrosis (46) and play a role in the process of fibrosis, such as in PF (47–50), renal fibrosis (51, 52), ischemic cardiac fibrosis (53, 54), and neovascularization (55).

Therefore, on one end of the extreme, M1 pro-inflammatory cells facilitate the eradication of infections, albeit with the potential to inflict damage. On the other extreme, M2 anti-inflammatory cells have a repair phenotype that promotes a regression phase of the injury response (51). In response to various signals, macrophages may undergo classical M1 activation (stimulated by TLR ligands and IFN-γ) or alternative M2 activation (stimulated by IL-4/IL-13). These states reflect Th1-Th2 polarization in T cells (56, 57). The M1 phenotype is characterized by high levels of pro-inflammatory cytokine expression, high production of reactive nitrogen and oxygen intermediates, promotion of the Th1 response, and potent bactericidal and tumoricidal activity (58). M1 macrophages are also believed to be involved in various chronic inflammatory and autoimmune diseases (59). M2 macrophages are considered to be involved in the control of parasites, promoting tissue remodeling and tumor progression, and have immunomodulatory functions. They exhibit effective phagocytic activity and high expression of scavenging molecules, among others (60).

#### 2.1.3 Function classification of homeostatic activities

Mosser and Edwards proposed a classification of macrophages based on three primary functions that these cells perform to maintain homeostasis in the body: host defense (classically activated), wound healing, and immune regulation (32).

##### 2.1.3.1 Host defense macrophages

The role of classically activated macrophages in host defense against intracellular pathogens has been well documented. Classically activated macrophages, as mentioned earlier, are crucial for host defense. However, their activation needs to be tightly regulated due to the potential for cytokines and mediators they produce to cause host-tissue damage. For instance, classically activated macrophages produce IL-1, IL-6, and IL-23, which have been associated with the development and expansion of TH17 cells (61). These cells produce IL-17, a cytokine involved in recruiting polymorphonuclear leukocytes (PMNs) to tissues, potentially contributing to inflammatory autoimmune pathologies. On the other hand, macrophages can inhibit inflammation by clearing apoptotic PMNs during inflammation, partly due to the production of TGF-β (62–64).

##### 2.1.3.2 Wound-healing macrophages

Macrophages play a vital role in wound repair (11, 65). Alternatively, activated macrophages have anti-inflammatory functions and are involved in regulating wound healing. They contribute to dampening inflammation, clearing cell debris, and coordinating tissue repair, making them essential for the wound healing process (66). Wound-healing macrophages can develop in response to innate or adaptive signals. IL-4, released during tissue damage, is one of the initial innate signals that rapidly convert resident macrophages into a population of cells programmed to promote wound healing (67). IL-4 stimulates arginase activity in macrophages, allowing them to convert arginine to ornithine, a precursor of polyamines and collagen that contributes to extracellular matrix (ECM) production (68). When the inflammatory stimulus or pathogen is eliminated, M1 cell activation diminishes. Alarmins and Th2-type cytokines drive the immune response toward a wound-healing response characterized by the accumulation of M2 macrophages. These M2 macrophages promote wound healing and fibrosis by producing matrix metalloproteinases (MMPs), including MMP12, tissue inhibitor of metalloproteinases 1 (TIMP1), growth factors (including platelet-derived growth factor (PDGF)) and cytokines (such as TGF-β1) (29).

##### 2.1.3.3 Regulatory macrophage

Regulatory macrophages have a key role in regulating the inflammatory immune response to limit tissue damage. Their primary physiological function is to dampen inflammatory immune responses and prevent the immunopathology associated with prolonged activation of classically activated macrophages (66). They are characterized by the production of high levels of IL-10 (69). Regulatory macrophages can secrete large amounts of this cytokine in response to Fc receptor γ -binding (34, 70). They represent a relatively broad category of macrophages that play a crucial role in inhibiting inflammatory immune responses and preventing the immunopathology associated with prolonged activation of classically activated macrophage (71). They are distinct from classically activated macrophages and differ from macrophages treated with Th2 cytokines, such as IL-4 or IL-13, known as alternatively activated macrophages (72).

#### 2.1.4 Other classifications

Apart from M1 and M2 macrophages, there are additional subpopulations of macrophages, including tumor-associated macrophages (TAMs), CD169 macrophages, and T cell receptor-positive (TCR) macrophages (73).

##### 2.1.4.1 TAM

Macrophages display plasticity, with their phenotype determined by their location and the physiological or pathological context. Classically activated macrophages (M1) and alternatively activated macrophages (M2) represent two ends of the macrophage phenotype spectrum (74). TAMs closely resemble M2 macrophages and are associated with the inhibition of anti-tumor immunity (75). Myeloid-derived suppressor cells (MDSC) are often associated with TAM and may serve as their precursors (32, 76). TAMs promote tumorigenesis, tumor growth, invasion, metastasis, and affect tumor metabolism through various mechanisms (77). Recent study indicated that TAMs have protumoral functions, indicating that they play a direct or indirect role in promoting tumor progression (78).

##### 2.1.4.2 CD169 macrophages

As a specific subpopulation of macrophages, CD169 macrophages have been recently studied in malignant tumors (79). Current research suggests that CD169 macrophages have inhibitory effect on tumors. CD169/Siglec1/sialoadhesin, a sialic acid-binding immunoglobulin-like lectin, is primarily expressed in metallophilic macrophages in the marginal zone of the spleen and macrophages in the subcapsular sinus and medulla of lymph nodes. In addition to their role in anti-infectious immunity, recent study has demonstrated the involvement of CD169 macrophages in tumor immunity and their association with a favorable prognosis (79).

##### 2.1.4.3 T cell receptor

The T cell receptor (TCR) is a molecule essential for antigen recognition and forms a complex with CD3 (80). Previous studies have reported the presence of TCR macrophages in both human and murine populations. TCR-αβ has been observed in peripheral blood monocytes and *in vitro* in activated monocyte-derived macrophages. TCR macrophages can release CCL2 and exhibit a high phagocytosis capacity (81). Recently, Fuchs et al. (82) reported that TCR-αβ macrophages are present in murine and human atherosclerotic lesions, indicating their potential as a novel molecular target for diagnosing and treating diseases where cholesterol plays a central role in the pathophysiology.

### 2.2 Macrophage function

Macrophages have highly diverse roles in maintaining the body’s integrity, including direct participation in pathogen elimination and tissue repair during aseptic inflammatory conditions. Their functions vary across different tissues, playing crucial roles in tissue development, immune response to pathogens, surveillance and monitoring of tissue changes, and maintenance of tissue homeostasis.

#### 2.2.1 Phagocytosis and elimination of pathogenic microorganisms

Macrophages are specialized phagocytes that, often with a long lifespan, are present in all organs to maintain tissue integrity, remove debris, and respond rapidly to initiate repair in the event of innate immunity after injury or infection (30, 83). Plasticity and functional polarization are the hallmarks of the mononuclear phagocyte system (41). Their phagocytic activity is crucial for fibrogenesis, with the type of engulfed dead cells influencing fibrosis progression (84). Macrophages also act as heterologous phagocytes, detecting pathogen-related molecular patterns and injury-related molecular patterns through pattern recognition receptors (85, 86). TAMs demonstrate bidirectional transformation between anti-inflammatory and immunosuppressive phenotypes (57, 87). Furthermore, macrophages play a vital role in wound repair (65).

#### 2.2.2 Antigen presentation, immunomodulation, and anti-inflammatory function

Macrophages have the capacity to take up and present antigens, bridging innate and adaptive immunity (88). They can act as antigen-presenting cells (APCs) and influence adaptive immune responses (89). Monocytes that enter the tissue during inflammation can carry antigens to lymph nodes and present them to naive T-cells (90). Regulatory macrophages have been shown to efficiently present antigens and induce antigen-specific T-cell responses dominated by the production of Th2 cytokines (89). Macrophages also play a crucial role in cellular immunity by secreting cytokines and chemokines, regulating the activities of other immune cells, and balancing the body’s immune response. They can secrete both pro-inflammatory cytokines, such as IL-1 and IL-6, to promote inflammatory reactions, and anti-inflammatory cytokines, such as IL-10, to inhibit excessive inflammation.

#### 2.2.3 Regulation function regulating fibrosis

Macrophages are considered to be the critical cell types in the development of fibrotic diseases (17). Recent studies have also revealed that their role as regulators of fibrosis. Like myofibroblasts, these cells are derived from resident tissue populations such as Kupffer cells or bone marrow migrants (91–95). Current studies have shown that the pathogenesis of fibrosis is tightly regulated by different populations of macrophages, which exert unique functional activities in the initiation, maintenance, and regression stages of fibrosis (96, 97). Activated hepatic stellate cells (HSCs) attract and stimulate macrophages, which produce profibrotic mediators like TGF-β1 and PDGF, directly activating fibroblasts (94, 98). Several studies have identified macrophages as a major source of TGF-β1 and PDGF in fibrosis (71, 99). While macrophages contribute to fibrosis progression, they may also mediate its regression (11). Given the multifunctional capacity and heterogeneous phenotype of macrophages, it is not surprising that they can enhance and limit fibrosis (100). M2 macrophages may be a promising potential target for future anti-fibrosis therapies.

## 3 Overview of myofibroblast

### 3.1 Source and characteristics of myofibroblasts

In 1971, Gabbiani and his colleagues discovered and characterized myofibroblasts, which are fibroblasts modified to exhibit active contraction in rat wound granulation tissue. This was the first time it had been shown that myofibroblasts promote dermal wound contraction (101). Myofibroblasts are a subset of activated fibroblasts that express molecular markers such as α-SMA and the fibronectin (FN) splice variant extracellular domain (ED)-A FN (102). Hyperactive myofibroblasts, marked by the expression of α-SMA, are primarily responsible for the production of pathogenic collagen tissue fibrosis (7, 103). One of the defining features of myofibroblasts is the development of *in vivo* stress fibers and contractile force (104). They exhibit morphological and structural characteristics similar to smooth muscle cells, including a flat and irregular morphology, developed cell-ECM interactions, and intercellular space junctions (105). The activation of myofibroblasts is crucial for physiological and pathological tissue repair. Myofibroblasts are the main ECM secretory cells in wound healing and fibrosis and are mainly responsible for the contractility of scar tissue when it matures (106). Myofibroblasts combine the ECM synthesis characteristics of fibroblasts with the cytoskeletal characteristics of contractile smooth muscle cells, regulating connective tissue remodeling (107).

Defining characteristics of myofibroblasts include abundant rough endoplasmic reticulum, moderately developed peripheral myofilaments with focal density, fibronectin, and α-SMA immunostaining (108). In wound granulation tissue, myofibroblasts coexist with prominent endoplasmic reticulum and contractile microfilaments (109). The transformation of myofibroblasts is triggered by integrating neurohumoral, cytokine, growth factor, and mechanical signals from the extracellular environment (110). Myofibroblast differentiation is a critical event for wound healing, tissue repair, and chronic fibrosis (104, 107, 111). At least three local events are required for the differentiation of α-SMA-positive myofibroblasts: accumulation of biologically active TGF-β1, the presence of specialized ECM proteins like ED-A splice variants of fibronectin, and high extracellular stress are caused by the mechanical properties of ECM and cellular remodeling activity (104). The mechanical resistance of the ECM, combined with the action of fibrotic TGF-β1, is the primary stimulus for the differentiation and persistence of myofibroblasts (104).

### 3.2 Distribution of myofibroblasts

Myofibroblasts can originate from various sources, including epithelial-mesenchymal transition (EMT) (7), endothelial-mesenchymal transition (112, 113), resident fibroblast or pericyte proliferation (114), and the newly discovered phenomenon of MMT (115). Experimental evidence demonstrates that about 50% of myofibroblast accumulation comes from local proliferation of resident tissue fibroblasts, while approximately 35% comes from bone marrow-derived cells (116). Bone marrow transplantation studies have demonstrated the ability of bone marrow-derived cells to populate distal tissue sites (115, 117, 118).

### 3.3 The hazards of myofibroblasts

Myofibroblasts pose hazards in various ways. They are the primary cells responsible for collagen production in tissue fibrosis, and their contraction and ECM remodeling activity play a crucial role in fibrotic diseases (119–121). The fate of myofibroblasts in injured tissues, regardless of their origin, may ultimately determine whether healing occurs normally or progress to end-stage fibrosis (107). Persistent myofibroblast activity leads to progressive tissue fibrosis and distortion of the typical tissue architecture, resulting in organ failure and, ultimately, death (89). While the high contractile force generated by myofibroblasts is beneficial for physiological tissue remodeling, excessive force can be detrimental to tissue function, as seen in hypertrophic scars, fibrotic diseases, and stromal reactions to tumors (111).

Myofibroblasts are also critical components of the matrix reaction around hepatocellular carcinoma, contributing to the extracellular matrix component (122, 123). Activated hepatic stellate cells, portal vein fibroblasts, and bone marrow-derived myofibroblasts have been identified as central collagen-producing cells in the damaged liver (91). They play significant roles in renal fibrosis and are implicated in its pathogenesis (124). Additionally, myofibroblasts contribute to chronic cardiac fibrosis (110). Experimental and clinical observations suggest that myofibroblasts produce pro-invasive signals that may be associated with cancer progression and pain (125). Myofibroblasts present in the matrix reaction of epithelial tumors may contribute to the progression of cancer invasion (126, 127).

## 4 The contribution of MMT to the pathogenesis of PF

### 4.1 Introduction of PF

PF is a chronic and progressive irreversible pulmonary interstitial disease that poses a significant public threat health (128). It is a characteristic feature of a large class of interstitial lung diseases (ILD) (129, 130). Symptoms of PF typically include shortness of breath, unproductive cough, weight loss, and fatigue due to hypoxia (131). It is characterized by thickened fibrotic alveolar walls leading to impaired gas transfer, restricted ventilatory patterns, and, as a result, respiratory failure (132, 133).

Pre-existing inflammation is a key factor in PF development. Acute lung injury (ALI) and its more severe manifestation, acute respiratory distress syndrome (ARDS), are specific forms of lung inflammation characterized by diffuse alteration of the alveoli, non-cardiogenic lung edema, and local and systemic inflammation (134–137). Inflammatory cascades contribute to the pathogenesis of ALI, resulting in increased permeability of lung capillary vessels and diffuse alveolar damage (138–140). The pathomorphological changes in the lungs during ALI/ARDS include neutrophilic inflammatory infiltration, diffuse alveolar damage, alveolar and interstitial edema, hyalin membrane formation in the exudative phase, and ECM deposition in the proliferative phase (139, 141, 142).

PF is a heterogeneous disease characterized by a distinct pattern of tissue pathology and comprises a large number of chronic respiratory pathologies accompanied by connective tissue growth in various lung compartments, among which interstitial lung disease (ILD) and idiopathic PF (IPF) are the most severe and irreversible ones with progressive fibrosing of the lung parenchyma (130, 143–145). IPF, specifically, is a significant type of pulmonary fibrosis, predominantly affecting the elderly, with high mortality and poor prognosis (146). It can cause dyspnea, cough, impaired lung function, and death (147–149). The prevalence of IPF is around 10 cases per 100,000 population, while ILDs have a prevalence of 19.4 cases per 100,000 population (150, 151). In 2014, two drugs, pirfenidone and nintedanib, were approved by the FDA for the treatment of PF (152). However, effective therapeutic options for PF are still lacking, and current treatments only delay disease progression without providing a complete cure. Moreover, these drugs have undesirable side effects, such as gastric and intestinal bleeding and severe diarrhea. Lung transplantation is the last resort for patients, offering some extension of lifespan, but it is not accessible to most individuals. Therefore, studying the molecular mechanisms underlying the transition from acute lung inflammation to PF and identifying new molecular markers and promising therapeutic targets for preventing PF development remain important objectives.

### 4.2 Role of macrophages in pulmonary fibrosis

Macrophages, as innate immune cells with antibacterial and phagocytic activity, play a significant role in PF. They are the most abundant immune cell population, accounting for about 70% (153). They are widely distributed in the lung and alveolar tissue and are involved in almost all the physiological and pathological processes of the lung (154). They are the host lung defense, indispensable paramount sentry (155, 156), and also play a vital role in the pathogenesis of PF. Macrophage infiltration is observed in PF (157). Macrophages are involved in all stages of lung injury and repair and can both promote and inhibit fibrosis. They play an essential role in the removal of lung pathogens clearance and maintaining homeostasis (157, 158). The pathogenic role of macrophages in PF has been investigated in multiple studies, involving reactive oxygen species generation (159–161), stimulation of proteinase-activated receptors (162, 163), and secretion of pro-fibrotic cytokines (164, 165).

There are three main types of pulmonary macrophages: alveolar macrophages (AM), interstitial macrophages (IM), and bronchial macrophages (BM), with AM accounting for more than 90% (166). Different subtypes of macrophages play distinct roles in lung injury, repair, and fibrosis (167). Single-cell sequencing of lung tissue from patients with PF have confirmed that alveolar macrophages play an essential role in PF (168–170). Alveolar macrophages are the first cells to come into contact with external pathogens and irritants, initiating and later resolving lung immune responses. Additionally, macrophages have other organ-specific functions, such as surfactant utilization and absorption of apoptosing and destroying cells (171–174). Monocyte-derived macrophages are key drivers of PF and supplement alveolar macrophages that are lost immediately upon injury (175, 176).

The effect of macrophages on PF is mainly related to their polarization, which occurs during the repeated damage and abnormal repair of alveolar epithelial cells (177, 178). Epithelial apoptosis is a critical component of fibrotic disease in many organs, including the lung (179, 180). Down-regulating the pro-fibrosis activity of alveolar macrophages or depleting this group of cells can effectively treat experimental PF (181–183). Macrophages can polarize into either a pro-inflammatory M1 phenotype or an alternatively activated M2 phenotype, depending on the microenvironment in which they reside (184). In response to lung injury, macrophages undergo a transition into pro-inflammatory M1 phenotypes and begin to secrete pro-inflammatory cytokines (TNF-α, IL-6, IL-1) and chemokines (IL-8, CCL7, CCL2), which leads to the increased chemotaxis and progressive enrichment of alveolar spaces by monocytes and neutrophils (185), which aggravate the pulmonary inflammatory response. On the other hand, M2 polarization releases various cytokines, such as TGF-β1 and IL-10, promoting the generation of myofibroblasts and the deposition of extracellular matrix, ultimately leading to PF.

**Figure 2:**
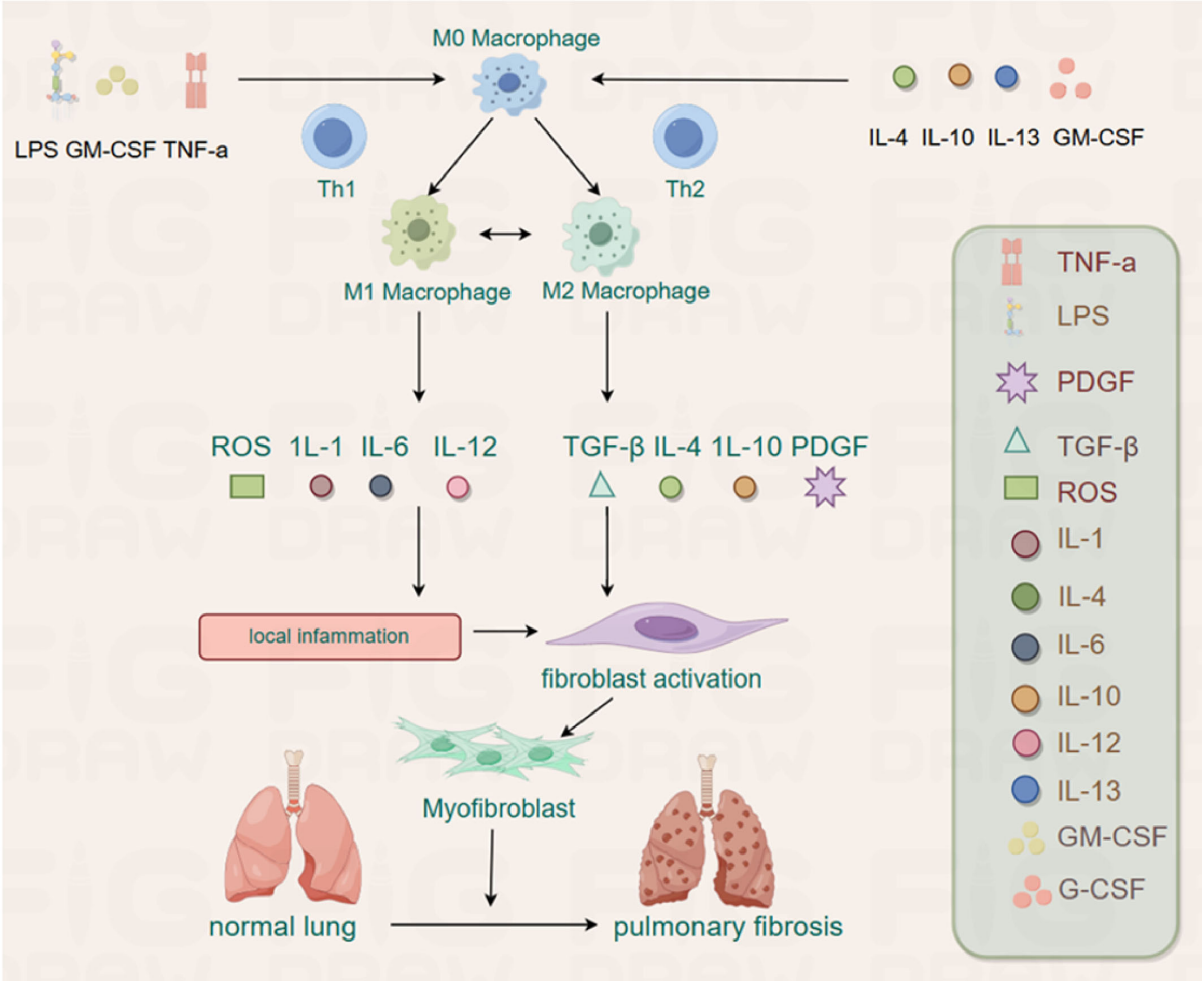
The M0 can be polarized into M1 and M2 by different stimuli. The M1 plays an inflammatory role by releasing ROS, IL-1, IL-6 and IL-12, whereas M2 has the potential to promote fibrosis by releasing TGF-β, IL-4, IL-10 and PDGF in PF.

During tissue damage and early inflammation stages, the activation of M1 macrophages promotes inflammation through extracellular matrix-degrading MMP and pro-inflammatory cytokines. An active cytokine environment, including Th1 cytokines, IL2, IFN-γ, and TNF-α, drives M1 macrophage activation. In contrast, other types of interstitial lung diseases (ILDs), including PF, often have a higher proportion of anti-inflammatory M2 macrophages (186) (**Figure 2**).

In the progression of PF, M1, and M2, macrophages are recruited to the site of the lung tissue injury site to regulate the fibrotic process after basement membrane destruction. M1 macrophages play a crucial role in matrix degradation by directly and indirectly producing MMP and various anti-fibrotic cytokines, essential for ECM remodeling and help reduce the pathological fibrous proliferation observed in late ALI (187). In contrast, M2 macrophages promote fibrous proliferation and ECM deposition in lung tissue (188, 189). Therefore, the degree of PF depends on the balance between M1 and M2 macrophages in the local microenvironment of lung tissue injury. Studies have shown that macrophages, predominantly M2 macrophages, contribute to the pathogenesis of PF (155, 190). M2 macrophages are the primary source of TGF-β1 and platelet-derived growth factors that induce fibroblast differentiation into myofibroblasts, initiating PF (191). Macrophage subsets may regulate fibrosis by differentiating into myofibroblasts, acting as sources of cytokines and growth factors with fibrotic properties, and secreting proteases involved in matrix remodeling (192). Therefore, the number and phenotype of macrophages are considered essential for the pathological process of PF (193, 194). While macrophages are essential for lung defense, they can also lead to tissue damage (195). Different subtypes of macrophages play distinct roles in lung injury, repair, and fibrosis (196).

### 4.3 Role of myofibroblasts in PF

The main morphological characteristics of PF, such as ECM deposition and remodeling of lung architecture, are consequences of a disbalance between two physiological processes in the lungs: (1) proliferation/apoptosis of fibroblasts and myofibroblasts; (2) synthesis/degradation of ECM components (197). These processes are closely interconnected, and the disruption of fibroblast and myofibroblast functioning is the primary driver behind the imbalance of ECM homeostasis and the development of PF. The fibroblastic phenotype present in that diseased lung primarily by the production of several soluble factors, such as TGF-β, PDGF, VEGF, and thrombospondin 1, which can differentiate resident fibroblast into myofibroblasts (170, 181, 195). Regardless of the source of lung fibroblasts, myofibroblasts, which resemble smooth muscle cells in terms of their contractile ability and expression of α-SMA, are considered the key cells in PF development.

Myofibroblasts are the primary effectors responsible for the excessive production of collagen and other extracellular matrix proteins in fibrotic lungs (104, 198). These contractile fibroblasts express α-SMA and abnormally proliferate in PF. They play a significant role in the occurrence and progression of PF by synthesizing and secreting large amounts of ECM components, such as collagen (I, III, IV, V, and VI), fibronectin, and laminin (199–201), making them critical in regulating the progression of PF. Myofibroblasts have also been found to secrete or release various proteins, lipids, and nucleic acid molecules that contribute to the pathological characteristics of other cell types in fibrotic lungs (129).

The accumulation of myofibroblasts is considered a marker of PF (202). Current research indicates that myofibroblasts involved in PF originate from several sources, including the proliferation and differentiation of resident fibroblasts, the recruitment of circulating fibroblasts to injury sites in organs, endothelial-mesenchymal transformation, and epithelial-mesenchymal transformation (203–205). The synthesis of pathogenic collagen by myofibroblasts, as the main effector of tissue fibrosis, and the process of MMT are essential regardless of the etiology of fibrosis (3, 206–208). Myofibroblast transdifferentiation is a marker of the fibrotic response. Evidence suggests that macrophages are involved in regulating fibrotic responses, with pulmonary myofibroblasts being the primary target for the development of new therapies for IPF (104, 198).

### 4.4 MMT related signaling pathways in the development of PF

**Figure 3:**
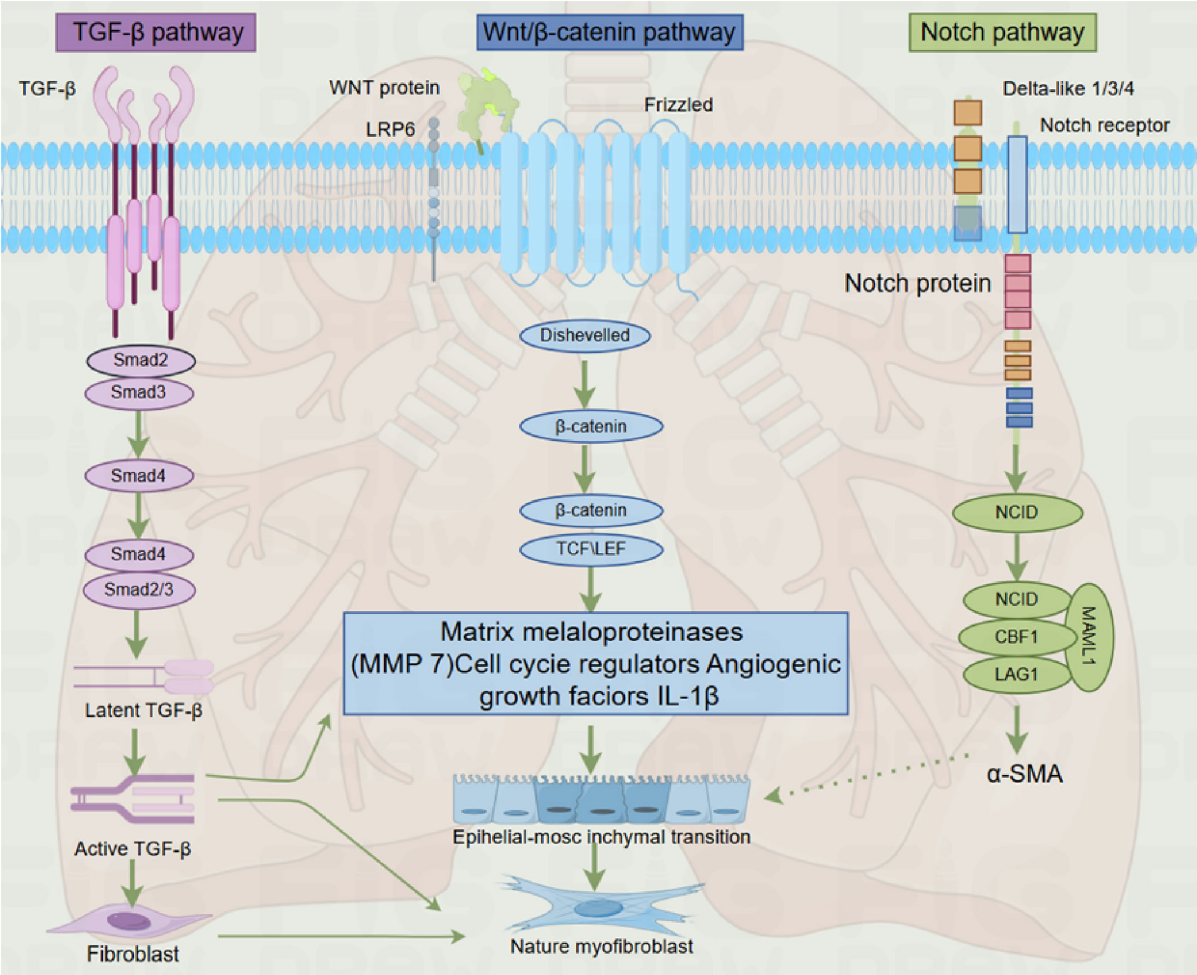
Overview of particular signaling pathways regulating pulmonary fibrosis development.

As described earlier, fibrosis is defined by the excessive accumulation of fibrous connective tissue in and around inflamed or damaged tissue, which can lead to permanent scarring, organ malfunction, and, ultimately, death, as seen in end-stage liver disease, kidney disease, IPF, and heart failure (91, 209). The development of PF involves genes and molecular pathways that primarily participate in pre- and postnatal lung development (210, 211). The key pathophysiological events of IPF include repetitive alveolar epithelial cell injury, the presence or absence of local inflammation, impaired epithelial-mesenchymal crosstalk, and subsequent fibroblast-to-myofibroblast activation (212–214). These mechanisms are mediated by abnormally activated signaling molecules that drive the process of fibrosis, such as TGF-β, Wnt/β-catenin, hedgehog, Notch, and fibroblast growth factor signaling pathways, with the TGF-β signaling pathway being the most critical (215, 216). While most of these pathways are inactive in the adult organism, they become active during tissue regeneration, and the chronic pathological activation of these signaling pathways is associated with injury restoration processes in all organs, including the lungs (210, 217, 218). Furthermore, a recent study demonstrated that nintedanib, one of the FDA-approved anti-fibrotic drugs, modulates TGF-β, VEGF, and Wnt/β-catenin signaling pathways, further supporting the central role of these pathways in PF development (219) (**Figure 3**).

#### 4.4.1 TGF-β pathway

##### 4.4.1.1 TGF-β biology

TGF-β is a member of a large polypeptide family, modulating several biological processes, including proliferation, differentiation, and cell apoptosis in internal organs (219). Initially isolated from platelets, TGF-β is a multifunctional cytokine that plays a crucial role in regulating fibrosis both at physiological and pathological levels (220, 221). The TGF-β signaling pathway is activated during the development of fibrosis in different tissues and regardless of the underlying cause. It leads to increased *de novo* synthesis of TGF-β by multiple cell types, including macrophages, platelets, and T-cells, as well as increased release from the extracellular matrix (222–225). Among the three identified members of the TGF-β family in mammals (TGF-β1, TGF-β2, and TGF-β3), TGF-β1 is the predominant form expressed in the immune system, and it is the most abundant subtype in most tissues, including the skin. TGF-β1 is a pro-fibrotic cytokine and a key initiator of organ inflammation and fibrosis (226–228). It can induce the differentiation of epithelial or endothelial cells into myofibroblasts *in vitro* (229–231).

##### 4.4.1.2 TGF-β/Smad pathway

The TGF-β/Smad pathway is the primary signaling cascade through which the TGF-β signal is transduced into various cellular responses. Smad proteins, a family of cytoplasmic signal transduction proteins, mediate the signals from activated TGF-β receptors and interact with TGF-β responsive promoters. Smad2 and Smad3 are the key mediators of signals from activated TGF-β receptors, and they form complexes with other transcription factors to bind to DNA and regulate gene expression (232). Classical TGF-β1 signal transduction operates through TGF-β receptors and Smad2/3/4 transcription factors (230). In the tissue fibrosis models, the protective effects observed in Smad3 gene knockout mice indicate that TGF-β/Smad3 signaling is pro-fibrotic, while conditional Smad2 deficiency promotes fibrosis, indicating the opposite effects of Smad2 and Smad3 (233–235). It has been demonstrated that Smad3 is a key signaling pathway for fibrosis both *in vivo* and *in vitro* (131, 236, 237). The key role of Smad3 in the development of fibrosis has also been reported in many disease models, including bleomycin-induced PF (234). The TGF-β signaling cascade involves the binding of TGF-β to its receptors (TGF-βRII and TGF-βRI), leading to the activation of Smad2 and Smad3, their translocation into the nucleus, and the transcription of target genes (238).

##### 4.4.1.3 Pathogenic effect of TGF-β in fibrosis

Macrophages are the primary source of the main effector molecule TGF-β in fibrosis. TGF-β is the primary effector molecule in fibrosis, promoting the proliferation of fibroblasts and collagen synthesis by producing growth factors, thereby promoting fibrosis (239). It accelerates the progression of PF by recruiting and activating monocytes and fibroblasts and inducing ECM production at the site of injury (240). Macrophages are one of the most important regulators of the fibrotic response, secreting cytokines, growth factors, and ECM-regulating proteins (43). They promote PF by releasing pro-fibrotic mediators (such as TGF-β), chemokines, and matrix metalloproteinases. TGF-β stimulates lung fibroblasts, circulating fibroblasts, and small airway epithelial cells to transdifferentiate into myofibroblasts (199).

TGF-β promotes fibrosis through various mechanisms, including the induction of myofibroblasts, increased synthesis of ECM components, and inhibition of collagen degradation (241). It plays a central role in the pathogenesis of PF by promoting the activation, proliferation, and differentiation of epithelial cells and collagen-producing myofibroblasts (242). TGF-β signaling is one of the most potent inducers of fibroblast activation, stimulating the synthesis of ECM components and inhibiting their degradation by matrix metalloproteinases (243, 244). It also regulates the differentiation of fibroblasts into myofibroblasts (245). TGF-β1, β2, and β3 are all involved in embryonic lung development, the maintenance of organ homeostasis, and responses to tissue damage. Increasing evidence suggests that the TGF-β pathway is activated in chronic lung diseases, including IPF (246). IPF and interstitial PF are particularly serious lung diseases, with TGF-β signaling pathway playing a significant role in fibrosis (247, 248).

#### 4.4.2 Wnt/β- catenin signaling pathway

The Wnt gene family consists of 19 secreted glycoproteins and is involved in the regulation of mammalian embryonic development and tissue regeneration, making up the Wnt signaling pathway (249). Classical Wnt signal transduction inhibits the phosphorylation of β -catenin in the cytoplasm and subsequent translocation into the nucleus and activation of the transcription factor TCF/LEF (250). The Wnt signaling pathway plays a vital role in the development and maintenance of multiple organ systems, including the brain, intestine, hematopoietic system, skin, and lung (251–253). Increasing evidence shows that the Wnt family of secreted glycoproteins and their associated signaling pathways are involved in the development and play an active role in wound repair and regeneration events, including PF, cancer, heart valve formation, and aortic valve calcification (217, 254–257).

Classical Wnt signal transduction regulates the expression of multiple gene families, including MMPs and angiogenic growth factors, which play a role in PF development (258, 259). Activation of the classical Wnt pathway is a common feature observed in fibrotic disorders, occurring in systemic fibrotic conditions like SSc and isolated organ fibrosis in the lung, kidney, or liver (19, 260–265). The data suggest that inhibition of the classical Wnt pathway may be an effective way to target TGF-β signaling in fibrotic diseases (266). Several Wnt genes, including Wnt2, Wnt5a, Wnt7b, Wnt11, and Wnt13, are expressed in developing and adult lungs (251). In the adult lung, the Wnt pathway maintains balance by regulating stem and precursor cells in both healthy conditions and during the response to injury (267).

Wnt/β-catenin signal transduction induces an anti-apoptotic and pro-fibrotic phenotype in lung fibroblasts, leading to fibroblast proliferation and differentiation into myofibroblasts, exacerbating lung tissue fibrosis (268). Activation of AEC II by Wnt/β-catenin increases the production of IL-1β, stimulating inflammatory and pro-fibrotic responses (269). Atypical activation of Wnt also stimulates fibroblast proliferation and increases the synthesis of ECM components (270). In adult lungs, the Wnt pathway maintains homeostasis by regulating stem and precursor cells, both in healthy conditions and during response to injury (267, 271). Additionally, Wnt signaling is involved in epithelial cell proliferation, EMT, myofibroblast differentiation, and collagen synthesis (217). In the epithelial cells of the lungs, Wnt stimulates the production of surfactant and AEC II into AEC I differentiation (272). In contrast, in lung fibroblasts, Wnt increases proliferation and fibronectin expression and inhibits apoptosis (270). Recent studies have also demonstrated the activation of Wnt signaling in IPF, suggesting that this pathway plays a role in the pathogenesis of human PF (19, 217). Inhibition of Wnt/β-catenin signaling leads to the neutralizing of bleomycin-induced PF (273). The Wnt pathway takes part in PF pathogenesis through multiple mechanisms, including: (1) Wnt/β-catenin signaling pathway induces the anti-apoptotic and pro-fibrotic phenotype in lung fibroblasts, leading to fibroblast proliferation and their differentiation into myofibroblasts, exacerbating lung tissue fibrosis (268). (2) Activation of AEC II by Wnt/β-catenin increases IL-1β production, stimulating inflammatory and pro-fibrotic responses (269). (3) Atypical activation of Wnt also stimulates fibroblast proliferation and increases the synthesis of ECM components (270).

Additionally, cooperative signaling pathways of Wnt/β-catenin and TGF-β play an essential role in the development of PF: TGF-β was shown to induce EMT synergistically with Wnt/β-catenin (274). These findings suggest that targeting the interplay between TGF-β and Wnt/β-catenin may be a promising therapeutic approach for PF. By inhibiting or modulating the cross-talks between these pathways, it may be possible to intervene in the pathogenesis of PF and potentially mitigate its progression.

#### 4.4.3 Notch signaling pathway

The Notch signaling pathway is composed of four members in mammalian cells (275). With the exception of Notch4, all genes have been shown to regulate myofibroblast differentiation (276–279). Notch1 and Notch3 are known to stimulate lung fibroblasts (280). Moreover, Notch2 inhibit TGF-β induced α-SMA and collagen I gene expression by down-regulating Notch3 in myoblasts in hepatic stellate cells (278, 281), while in alveolar epithelial cells, Notch1 induces phosphorylation of Smad3 and activates α-SMA gene transcription in a manner dependent on SRF binding sites and TGF-β control elements (282). Other experiments have also shown that Notch1 inhibits fibroblast proliferation dependent on Wnt11-dependent WISP-1 expression (283). Notch signal transduction in fibrosis (including scleroderma (284)), may be due to the activation of this signaling pathway for myofibroblast differentiation, including through EMT)and endothelial-mesenchymal transformation.

The Notch signaling pathway is highly conserved and plays a crucial role in embryonic development and the homeostasis of various organs, including the lungs (285). It functions through paracrine signaling and one-way transmembrane receptors, regulating cell development during organogenesis. In adult lungs, along with other signaling pathways, the Notch pathway regulates stem cell functions and wound healing (285, 286). Enhanced Notch signaling has been observed during the development of PF (287), and the suppression of JAG1, Notch1, NICD, and Hes-1 has been shown to mitigate bleomycin-induced PF (288).

### 4.5 Effects of MMT on PF

MMT has been shown to contribute to interstitial fibrosis in patients with chronic renal allograft injury, a mouse model of unilateral ureteral obstruction (UUO), and progressive chronic kidney disease (1). Macrophages expressing CD68+ and α-SMA+ markers play a significant role in collagen production, particularly collagen I, and are associated with lung injury and interstitial fibrosis (12, 196, 289). MMT cells with M2 phenotype have been found to contribute to PF in animal models, including the lungs of rats with unilateral ureteral obstruction (UUO) (1, 196, 289). Eplerenone reduced the accumulation of MMT cells in the lung. In UUO rat lung fibrosis, UUO-induced lung injury, and fibrosis, MMT cells were found to account for the myofibroblast group, confirming that MMT plays a role in PF. These MMT cells in the lung exhibited an apparent M2 phenotype, indicating that the MMT process may be an important pathway leading to PF (12).

MMT plays a crucial role in the progression of chronic inflammation to pathological fibrosis, and the severity of interstitial fibrosis is closely related to the number of MMT cells (1, 51, 196, 289). MMT contributes to an increase in the population of myofibroblasts in the lungs, which have a strong proliferative capacity and further promote the proliferation of fibroblasts. Myofibroblasts, a subset of activated fibroblasts, are primarily responsible for organ deformation by inducing the deposition of fibrous collagen during tissue fibrosis (290). Upon transdifferentiation, myofibroblasts secrete various components of the extracellular matrix, including collagen, leading to excessive deposition of extracellular matrix in the lungs, a key pathological characteristic of PF. This excessive deposition disrupts the normal alveolar structure, resulting in alveolar collapse and reduced lung function.

The pro-fibrotic cytokine TGF-β1 is an essential initiator of organ inflammation and fibrosis by activating the downstream Smad signaling cascade, especially the Smad3 signaling cascade (6). Smad3 is a crucial transcription factor for classical TGF-β1 signal transduction (234, 291). The inhibition of MMT by targeting cytokines such as TGF-β1 or blocking the Smad3 signal pathway can slow down the process of PF. Moreover, the non-receptor tyrosine kinase Src, which can be activated by TGF-β1, has been closely associated with tissue fibrosis. Inhibition of Src has been shown to block MMT in animal models and reduce the severity of PF induced by bleomycin (292–294). However, further research is needed to fully understand the role of MMT in Src-mediated PF and explore the potential of Src-targeted therapy for blocking MMT and treating PF.

In summary, MMT plays an essential role in the process of PF, which accelerates the process of PF by promoting the transdifferentiation of macrophages into myofibroblasts. Inhibiting the MMT process represents a potential therapeutic target for anti-fibrotic treatment. Future studies should focus on elucidating the regulatory mechanisms of MMT and its specific role in PF to provide novel insights and treatment strategies for PF. A comprehensive treatment approach considering various factors, including inflammation control, inhibition of the fibrotic process, and improvement of lung function, is essential for effectively managing PF.

### 4.6 Effects of MMT on lung cancer

Lung cancer is the leading cause of death worldwide. For decades, it has remained the second most common cancer and the leading cause of cancer deaths, accounting for about 11.4% of new cancer cases and 18% of cancer deaths globally in 2020. Cancer‐associated fibroblasts (CAFs) are essential in tumor microenvironment (TME) driven cancer progression. CAFs are the most prominent stromal components (295). CAFs, a subtype of myofibroblasts, contribute to the malignancy and advancement of cancer (296). Cancer cells possess heterogeneity, versatility, and adaptability, resulting in primary and secondary drug resistance (297). The degree of macrophage-myofibroblast transition (MMT) has been found to be closely associated with the prognosis of certain cancers (297). MMT is an essential source of CAFs in non-small cell lung cancer (NSCLC). The hematopoietic transcription factor Runx1 has been identified as a critical regulator of MMT in cancer patients. Inhibition of Runx1, macrophage-specific and systemic, effectively blocks MMT-driven tumor formation *in vivo*, making it a potential therapeutic target for eliminating pro-tumor CAFs in patients with NSCLC (298).

Myofibroblasts can secrete various growth factors and cytokines, such as TGF-β and PDGF, which can stimulate the proliferation and migration of tumor cells and promote the progress of cancer. The TGF-β/Smad3 signal pathway is a critical regulatory factor promoting tumor microenvironment (299–301). It is essential to initiate MMT in chronic inflammatory diseases, including cancer. The MMT process and tumor growth in lung cancer are tightly regulated by Smad3 (302). TGF-β/Smad3 signal transduction is a key regulatory factor in the tumorigenic microenvironment. Recent evidence indicates that TGF-β can trigger the M1/M2 polarization of TAMs by activating Smad2/3 and PI3K/AKT pathways, thus enhancing the transcription of tumorigenic effectors such as IL-10, VEGFA, and CXCR4 (303). However, targeting Smad3 also inhibits T cell anti-cancer immunity, highlighting the complexity of potential therapeutic strategies (5, 207, 293, 304).

MMT is a critical pathophysiological process within the tumor microenvironment, leading to the generation of myofibroblasts that secrete inflammatory factors and fibrosis-related proteins in tumor tissues, promoting inflammation and fibrosis changes in the tumor microenvironment (305). Co-expression of TAM markers (CD68) and CAF markers (α-SMA) has been observed in lung, renal, and prostate cancers, indicating the presence of MMT in these types of cancer (1, 2, 196, 301). An interesting phenomenon in MMT is the further differentiation of TAMs into CAFs. Silencing Smad3 specifically in macrophages effectively inhibits MMT and consequently impedes CAF-mediated cancer progression. These findings highlight the significance of macrophage Smad3 in regulating CAFs through MMT, providing a specific therapeutic target for cancer immunotherapy (5). Given the critical role of MMT in cancer progression, inhibiting MMT may become a new target for cancer treatment. By blocking the process of MMT, the support of the tumor microenvironment can be weakened, the proliferation and migration of cancer cells can be inhibited, and the prognosis of cancer can be improved. Therefore, it is significant to study the mechanism and intervention strategy of MMT for developing new cancer treatment methods and improving cancer prognosis.

## 5 Summary and prospect

Organ fibrosis is a common pathway by which various chronic diseases progress to an end-stage state. The conversion of MMT is a process where bone marrow-derived macrophages differentiate into myofibroblasts, promoting organ fibrosis during injury. This paper reviews the origin, distribution, and characteristics of macrophages and myofibroblasts in organ fibrosis, along with their pathological effects on diseases caused by organ fibrosis. The purpose is to further understand MMT and its signaling pathway and to determine a new target for organ fibrosis treatment.

Current research on MMT primarily focuses on renal fibrosis, with limited studies on fibrotic diseases in other organs. The mechanisms and influencing factors of the conversion of MMT still require deeper exploration. Under specific conditions, MMT provides new ideas and possibilities for treating kidney, lung, and liver diseases. Future studies need to focus on the crucial role of the TGF-β/Smad3 signaling pathway in the progression of MMT and organ fibrosis. Targeting the TGF-β/Smad3 signaling pathway for MMT treatment is expected to become a viable strategy for the prevention and treatment of progressive fibrosis.

The discovery of the MMT process also provides a new direction for studying the possible mechanisms by which macrophages promote fibrosis and offers a basis for intervening in myofibroblast activity through multiple pathways. MMT not only serves as a new therapeutic target for the prevention of fibrotic diseases but also acts as a key checkpoint for the development of chronic inflammation into pathogenic fibrosis. Understanding and elucidating the phenomenon of MMT and its potential signaling pathways will aid in identifying therapeutic targets for fibrosis.
